# Application of Machine Learning Approach to Classify Human Activity Level Based on Lifelog Data

**DOI:** 10.3390/s26051612

**Published:** 2026-03-04

**Authors:** Si-Hwa Jeong, Woomin Nam, Keon Chul Park

**Affiliations:** 1 Energy Solution R&D Center Hanwha Ocean Co., Ltd., 14 Sejong-daero, Jung-gu, Seoul 04527, Republic of Korea; jsh67@hanwha.com; 2 Industry-Academic Cooperation Foundation, Tech University of Korea, Siheung 15073, Republic of Korea; applewoods9559@tukorea.ac.kr; 3 Business Administration Department, Tech University of Korea, Siheung 15073, Republic of Korea

**Keywords:** healthcare, lifelog, classification, machine learning, wearable devices

## Abstract

The present paper provides a human activity-level classification model based on the patient’s lifelog collected from wearable devices. During about two months, the heart rate, step count, and calorie consumption for a total of 182 patients were collected from a wearable device. Using the lifelog data, the machine learning models were developed to classify the physical activity status of patients into five levels. Three types of wearable data with heart rate, step count, and calorie consumption were pre-processed as integrated data in time series. A total of 80% of the integrated data was used as the training dataset, and the remaining 20% was used as the test dataset. Sixteen algorithms were evaluated, including 12 traditional machine learning models (SVM, KNN, RF, etc.) and 4 deep learning models (CNN, RNN, etc.), and cross-validation was performed by dividing the training dataset into 5 folds. By changing the parameters required for training, the models with optimal parameters were derived. The performance of the final models with the new patient lifelog data was evaluated, and it was shown that the classification for human activity level based on heart rate and step count can be performed with high accuracy.

## 1. Introduction

With the recent development in information and communication technologies (ICT), such as artificial intelligence and big data, the value of innovative convergence has become more important [1,2,3,4,5]. Owing to the rapid paradigm change and the COVID-19 pandemic, interest in medical examinations and disease prediction services without external contact is increasing in the healthcare fields [6,7,8,9,10]. Given that lifelogs are closely related to health and disease, it is very meaningful to define this correlation in the healthcare field [11,12]. Some studies have shown that lifelogs help doctors better understand patients and provide them with knowledge about their health, which can positively affect future disease treatment [13,14].

Lifelog is a compound word of ‘life’ and ‘log’, meaning daily and record, respectively, which refers to all information about human activities recorded in daily life. With the recent and more precise development of smartphones and wearable devices, lifelogs have been increasing exponentially. In addition, as ICT develops and the culture of sharing information and knowledge is established throughout society, many people are producing various types of lifelogs. In the medical field, data closely related to health, such as heart rate, step count, sleep time, blood sugar, and meal records, are utilized for medical purposes, and the lifelog type may vary depending on the status of the patient.

Although high-quality lifelog collection has become possible owing to the development of wearable devices, it remains limited in most cases as only simple records and statistical analysis are presented. For effective health management, it is necessary to analyze the patient’s condition based on the collected lifelogs. However, a critical research gap persists in the existing human activity recognition (HAR) literature, which predominantly targets general populations and often overlooks the integration of specific clinical phenotypes such as hypertension or diabetes [15]. This study advances prior work by bridging the gap between physiological signals and clinical backgrounds while addressing the ‘phantom sedentary’ problem through a dedicated not-worn classification. Conventional activity recognition models often focus on general performance for the general public, neglecting the specific physiological variations and clinical backgrounds of patients [16]. The primary research question of this study is how to develop a highly reliable and interpretable classification model by integrating physiological signals from wearables with the clinical characteristics (e.g., hypertension, diabetes, and dyslipidemia) of actual patients. To address this issue, the present study proposes a patient-specific lifelog analysis process. This approach specifically aims to optimize the balance among performance, interpretability, and efficiency, aligning with the need for personalized modeling in clinical HAR [17]. Instead of pursuing a general-purpose model, the goal is to develop a system tailored to patient populations by combining heart rate and step count data with their specific clinical backgrounds. To ensure data reliability, the patient’s physical activity status was categorized into five levels within the time series, including a newly defined ‘not worn’ state (Level 0). This dedicated classification addresses the ‘phantom sedentary’ problem by preventing false sedentary assignment, a technique essential for maintaining data integrity in clinical monitoring [18].

The machine learning models used included sixteen algorithms, consisting of twelve traditional machine learning models (support vector machine, k-nearest neighbors, random forest, naïve Bayes, decision tree, gradient boosting machine, XGBoost, etc.) and four deep learning models (Convolutional Neural Network, Recurrent Neural Network, Long Short-Term Memory, Gated Recurrent Unit). The parameter that produced the most optimal cross-validation result was identified through a process of model parameter tuning. The final machine learning models were selected by comparing the performances of the models with the optimal parameters applied. The model’s performance was evaluated using the following metrics: accuracy, recall, precision, and F1 score. This systematic evaluation aims to identify models that balance high accuracy and interpretability effectively. This balance is vital because high accuracy ensures patient safety, while interpretability allows medical practitioners make informed clinical decisions. When the final models were applied to the new patient lifelogs, it was confirmed that human activity levels were classified with an accuracy of over 93%, with top-performing models such as XGBoost and RNN exceeding 94%. This study proposes optimal machine learning models to classify human activity levels specifically tailored to the clinical characteristics of patients based on lifelogs. The remainder of this paper is organized as follows. Section 2 discusses related work. Section 3 discusses the materials and methodology, including data collection and pre-processing, machine learning algorithms, and performance. Section 4 analyses the results of the machine learning models to derive the optimal model and describes the results of applying it to real patient data. Finally, Section 5 provides a discussion, and Section 6 presents the conclusion of the study.

## 2. Related Work

In recent years, many studies have been conducted on healthcare services based on machine learning and artificial intelligence algorithms with smart devices that gather user lifelogs. Choi et al. proposed an intelligent healthcare service for the health status of an individual through data extraction, pattern analysis, and health life ontology modeling using smart device-based health lifelog analysis [19].

Building on existing healthcare informatics frameworks, prior HAR research can be broadly categorized into three streams: (i) traditional machine learning approaches (e.g., SVM, Random Forest, Naïve Bayes, KNN), (ii) deep learning approaches (e.g., Convolutional Neural Networks (CNNs), Recurrent Neural Networks (RNNs), Long Short-Term Memory Networks (LSTM), Gated Recurrent Units (GRUs)) that excel at extracting complex sequential patterns and hierarchical feature representations, and (iii) hybrid IoT-integrated architectures incorporating multimodal sensor fusion, distributed cloud computing, and interpretable AI methodologies for real-time healthcare applications [6,10,16].

While deep learning-based HAR studies, particularly CNN and LSTM architectures, have demonstrated superior classification performance in characterizing complex spatiotemporal activity patterns, especially in large-scale datasets collected from multimodal sensors [7], such approaches typically require significant computational resources and large annotated datasets. In contrast, traditional ML models remain highly effective for structured physiological and behavioral data such as heart rate and step counts. The former ensures robust performance with reduced complexity and high interpretability, whereas the latter often sacrifices interpretability for accuracy and scalability [20,21].

In consideration of the aforementioned factors, the present study is positioned within the initial category, undertaking a systematic comparison of widely adopted machine learning (ML) algorithms for wearable-derived lifelogs. The integration of heart rate and step count data is intended to demonstrate that meticulously calibrated machine learning models can attain competitive classification performance while preserving interpretability and practical applicability in healthcare contexts.

This research proposed an extraction method with pattern analysis on lifelogs and provided customized feedback to users. The average performance showed a 0.75 precision rate. Nam et al. presented a personalized healthcare service based on the lifelog information of a home resident obtained from a smart home system [22]. An accuracy of 0.88 was achieved by applying the NB algorithm, an activity recognition module for classifying user behavior. Kim et al. identified lifelog variables that had a decisive influence on body mass index (BMI) using gait and sleep information [20]. Variables associated with changes in BMI were also analyzed and characterized. This paper described that although wearable devices have advanced, because the accuracy of sleep data may be low due to external factors such as batteries and Wi-Fi, various pre-processing methods to improve the quality of sleep data and related variables were presented. Data were compared using ridge regression, XGBoost, and CatBoost, which are representative machine learning regression models, and the predicted results were interpreted by combining the machine learning model and SHapley Additive exPlanations (SHAP). Šabić et al. proposed a model for detecting heart rate abnormalities using a machine learning algorithm [21]. Heart rate data are important lifelogs because they can represent the physical status and potential diseases in an individual. Five machine learning algorithms were considered: three supervised and two unsupervised algorithms. These algorithms were evaluated using real heart rate data, and an abnormal heart rate was confirmed. Awotunde proposed a disease diagnosis system based on machine learning and wearable body sensors [23]. This system can be used for automated disease monitoring, diagnosis, prediction, and treatment. It provides fast, accurate, and reliable diagnostic results at a low cost. Data obtained from wearable sensors, such as body temperature and heart rate, were transmitted to the cloud database through the Internet of Things (IoT) devices. Machine learning was used to select the most useful features from the data, and the condition of the patient was diagnosed using machine learning. Evangelos et al. proposed a machine learning model to predict sedentary behavior based on step counts [24]. Four machine learning algorithms were discussed by collecting and analyzing data from 33 participants using wearable activity trackers. The models reviewed were logistic regression, RF, XGBoost, and Convolutional Neural Networks. The model performance evaluation showed an accuracy of approximately 0.8 and 0.74 for the existing and new datasets, respectively. Beyond these individual achievements, current research has found a structural gap between raw sensor accuracy and the dependability of clinical data. Recent studies suggest that most models are still susceptible to ‘compliance bias’, a phenomenon where times of device non-usage are often misclassified as sedentary activity [15]. This difference is especially noticeable in studies that rely only on heart rate and step count as the main measures, without a dependable method to detect non-wear periods. Achieving a balance among high-accuracy classification, medical interpretability, and clear identification of not-worn states marks a major step forward from basic activity models.

## 3. Materials and Methods

**Figure 1:**
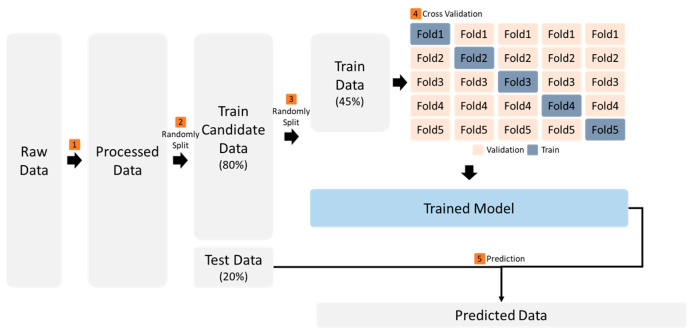
Overall process of the proposed method.

This section describes the research process and methodology of this study. First, the data types and the methodologies for data collection from the wearable devices are described. Given that the raw data obtained must be modified in a format suitable for machine learning, the data pre-processing adopted is described. The processed data input into various machine learning models and the optimal model derived through comparison and performance evaluation are described. Finally, the indicators used to evaluate the performance are described. The overall process adopted is summarized in Figure 1.

### 3.1. Ethical Statement

All procedures conducted with participants complied with the ethical standards of the institutional and national research committee and the Helsinki Declaration. Participants were informed about the study’s purpose and content and provided written informed consent for data collection and usage. The study results were anonymized to ensure participants’ privacy.

### 3.2. Participants

The lifelogs of 182 patients were gathered for approximately two months. The patients wore a Fitbit inspire2 device, the most common wireless physical activity tracker, on their wrists to continuously collect physical activity data, heart rate, step count, and calorie consumption in a free environment. Patients were diversely distributed regardless of sex and age, with the youngest being 19 years old and the oldest being 64 years old. All participants were trained in the use of wearable devices, the installation of apps on smartphones, and how to synchronize the collected information. They periodically synchronized the data with the Fitbit server, and the lifelogs were gathered by accessing the Fitbit server.

### 3.3. Data Collection

In this study, lifelogs were collected using Fitbit, one of the most commonly used wearable devices in the medical and healthcare industries. The performance of a Fitbit device has been verified in terms of accuracy and reliability [25,26].

The per minute heart rates of the patients were recorded using the Fitbit device. When the heart beats, capillaries expand and contract according to changes in the blood volume. To define the heart rate, the optical heart rate sensor of the device flashes a green LED repeatedly per second and uses a light sensitive photodiode to detect volume changes in capillaries above the wrist [27,28]. Green LEDs were used to maximize the signals from capillaries near the skin surface. Based on this principle, the Fitbit determines the heart beats per minute (BPM). Many factors can affect heart rates, such as ambient temperature, stress, caffeine and alcohol intake, exercise, and drugs; however, data collection has no restrictions. Consequently, approximately 86,400 heart rate data points were obtained per patient for two months. Approximately 15,724,800 heart rate data points were obtained for all patients.

The step count is calculated using a 3-axis accelerometer in the Fitbit device, which interprets the frequency, duration, intensity, and pattern of the movements of the user [29]. As the condition of the patient may be subject to variation, slight discrepancies may be observed in the number of steps recorded, contingent on factors such as gait speed and the nature of the activity (i.e., jogging). However, the findings of a preceding study indicate that the margin of error is deemed to be acceptable [25]. In addition, it was unnecessary to consider the effect of wearing the device on the right or left wrist; thus, the wearing conditions had no restrictions. The step count was automatically updated every 15 min. The value recorded at 15-min intervals was the number of steps added from 15 min before and not the accumulated number of steps. The recorded step count per day was 96, and since lifelogs were collected for approximately two months, approximately 5760 steps per patient and 1,048,320 step count data for all patients were obtained.

**Figure 2:**
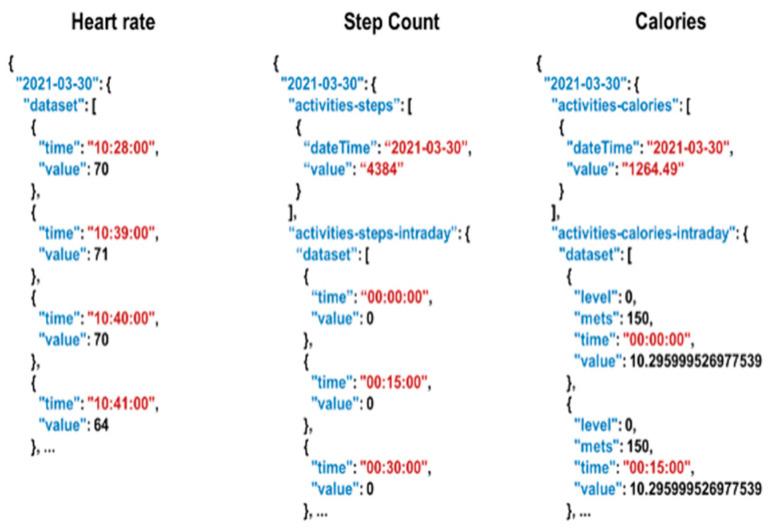
Raw data gathered by Fitbit API.

In the Fitbit device, calorie consumption information is estimated by combining activity data with the basal metabolic rate (BMR), which is the rate of burning calories to perform basic, life sustaining functions such as breathing, blood circulation, and heartbeat. The calories consumed during exercise were estimated using heart rate data. BSM was defined based on body data saved in individual Fitbit accounts, such as height, weight, sex, and age, and generally accounts for 60–70% of the total calories burned in a day. Calorie values were updated and recorded every 15 min on the device. Furthermore, Fitbit classifies physical status into four levels according to activity status based on comprehensive records, including calorie information: sedentary, lightly active, fairly/moderately active, and very active. The raw data format gathered by the Fitbit device is illustrated in Figure 2.

### 3.4. Data Pre-Processing

To ensure reproducibility, the pre-processing stage involved refining an initial set of 22 engineered features into a final 15-feature vector optimized for machine learning. Comprehensive pre-processing of raw lifelog data from wearable devices led to the creation of a unified dataset for machine learning. The selection of these 15 final features was made with a view to represent demographic, physiological, clinical and temporal dimensions whilst concomitantly ensuring the removal of redundant or low-variance signals (see Table 1). The vector included standard lifelog features such as the normalized heart rate and step counts. Moreover, additional features comprised temporal variables including time of day (Morning 25.6%, Afternoon 24.0%, Evening 24.0%, Night 26.4%), day of week, and weekend status. The dataset also included age (20–63 years, mean 35), gender (123 female, 59 male), drinking, smoking and comorbidities (HTN, DM, DL). The target variable depicts the different activity intensity levels for five classes (Levels 0–4).

**Figure 3:**
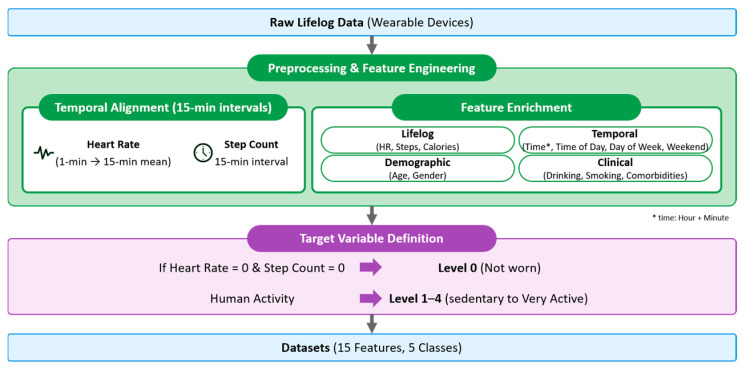
Data integration process.

To ensure that the cycle period of all the data was the same, the heart rate value recorded in a 1-min interval was converted into the 15-min interval form. The 15 values recorded for 15 min were averaged to obtain the mean heart rate over a 15-min period. The step count used were values recorded every 15 min without changing the existing data format. Calorie consumption information was recorded as a physical status classified into four levels according to the calories consumed at 15-min intervals. The target variable was categorized into five levels (Levels 0–4), where Level 0 (not worn) was explicitly defined when both heart rate and step count were zero to prevent false sedentary classification. However, it is necessary to define certain conditions when the device is not worn or does not work because of problems such as battery issues. If the device did not record data, the heart rate and step count were recorded as zero. Thus, the body status when the heart rate and step count were recorded as 0 was newly defined as Level 0 (not worn). The four levels in the Fitbit device were defined as Level 1 (sedentary), Level 2 (lightly active), Level 3 (fairly/moderately active), and Level 4 (very active). Accordingly, the human activity level defined in this study was divided into five levels: Level 0 to Level 4. In addition, Level 0 (not worn) was defined when both heart rate and step counts were zero. The complete pre-processing workflow and updated data integration flow is presented in Figure 3. Figure 3 shows the data integration process through pre-processing.

### 3.5. Machine Learning

This study proposes a machine learning algorithm that classifies physical activity status into five levels using the heart rate and step count data collected over a 15-min cycle. The researchers compared various traditional machine learning algorithms and deep learning algorithms to achieve optimal performance. To effectively capture both temporal dependencies and clinical contexts, we implemented a comprehensive suite of sixteen algorithms. The classification was performed using a 15-feature vector as input, with the output corresponding to the five activity levels. This included twelve traditional machine learning models (e.g., SVM, KNN, Random Forest, XGBoost, and ensemble methods) and four specialized deep learning architectures: CNN, RNN, LSTM, and GRU. Unlike traditional models that process static feature vectors, the deep learning models were designed to handle a three-dimensional input shape of (24, 15), corresponding to 24 hourly time steps across 15 integrated features. Machine learning input data consisted of a 15-feature vector including temporal, physiological, demographic, and clinical features. The output data corresponded to the human activity divided into five distinct levels.

#### 3.5.1. Algorithm

To improve the quality of machine learning, it is necessary to utilize data suitable for training. The total dataset of 182 patients was first divided into a training candidate group of 80% (146 patients) and a test group of 20% (36 patients) to prevent data leakage. Within the training candidate pool, we performed a secondary refinement to select 82 patients (45% of the total) who provided high-quality lifelogs. These participants provided sufficient heart rate and step counts, while 64 patients were excluded due to excessive ‘not worn’ (Level 0) data. This selection process was essential to prevent the model from over-fitting. Class 0 represents a deterministic pattern characterized by the absence of signals, yielding a near-perfect F1-score of 1.00. An abundance of theses trivial samples would hinder the model’s ability to learn the complex transitions between Levels 1–4. To ensure this refinement did not introduce selection bias, we compared the included (n = 82) and excluded (n = 64) participants (Table 2). Statistical tests indicated no significant difference in demographic and clinical variables (*p* > 0.05). Observed differences in physiological signals were methodologically consistent with the exclusion of low-quality data.

**Figure 4:**
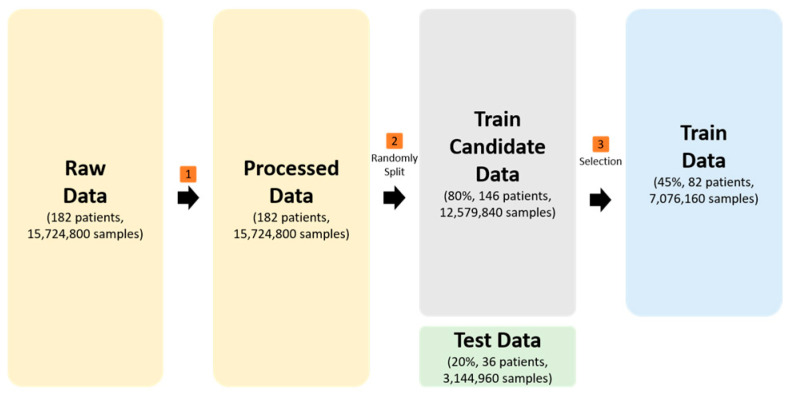
Dataset classification.

Consequently, the final training dataset consisted of 7,076,160 high-quality samples derived from 82 patients. This final cohort was verified to be demographically and clinically representative of the original candidate group. The dataset comprising 15,724,800 data points from 182 patients was split using a participant-wise approach to prevent data leakage. The test dataset, used for final model evaluation, contained 3,144,960 samples corresponding to 20% of the total data. The data classification process is summarized in Figure 4. The training and testing of the models was conducted utilizing the scikit-learn V1.7.2 library, which is an open-source Python library for machine learning algorithms, in conjunction with the PyTorch V2.6.0 deep learning framework. To ensure model stability and experimental transparency, all deep learning models were trained for a maximum of 100 epochs. The training process incorporated an early stopping mechanism, which halted training if the validation loss did not improve for 10 consecutive epochs. With regard to the machine learning models, the following methods were considered: support vector machine (SVM), k-nearest neighbor (KNN), random forest (RF), naïve Bayes (NB), gradient boosting machine (GBM), and XGBoost. The ensemble methods that were considered were as follows: Hard Voting and Soft Voting. The deep learning models were constructed with particular layers to ensure high-dimensional feature extraction and reproducibility. The CNN model used three 1D convolutional blocks, with filters increasing from 64 to 256, to extract local temporal features. This process involved the utilization of GELU activation and Batch Normalization. The recurrent models (RNN, LSTM, and GRU) had a three-layer stacked architecture with hidden units of 128, 64, and 32, to model long-term sequence dependencies within the lifelog data.

All deep learning models were integrated with a shared multi-layer perceptron (MLP) backbone. This backbone consisted of five fully connected layers, with dimensions ranging from 1024 to 256 units. To enhance the training process and promote the transfer of its benefits to other tasks, the Adam optimizer was used, with a learning rate of 0.002 and a batch size of 256. To mitigate overfitting, regularization was applied through Dropout (0.2–0.5) and Label Smoothing (0.1). Additionally, a learning rate scheduler reduced the learning rate by a factor of 0.5 whenever the validation loss plateaued for five epochs.

#### 3.5.2. Optimal Algorithm Selection and Hyperparameter Tuning

**Figure 5:**
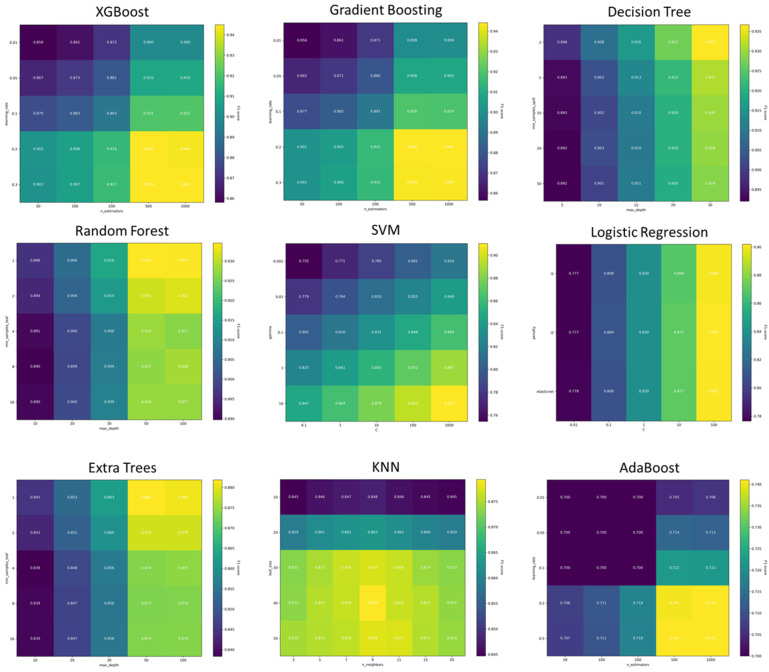
Hyperparameter tuning process.

To maximize the predictive performance of each model, we performed a randomized search over 20 iterations and 5-fold cross-validation. Figure 5 illustrates the hyperparameter tuning process, providing visual evidence of the model sensitivity to various parameter combinations. As shown in the heatmaps, several important parameter ranges included: SVM (C: 0.1–100, gamma: scale/auto/0.001–1), Random Forest (n_estimators: 50–300, max_depth: 10–30), XGBoost (n_estimators: 50–200, learning_rate: 0.01–0.2, max_depth: 3–7), etc.

This systematic approach enabled the identification of optimal parameters that balance accuracy and stability, as summarized in Table 2.

### 3.6. Model Evaluation

The model’s performance was evaluated based on four indicators: accuracy, precision, recall, and F1-score. Accuracy is an indicator of the amount of predictive data that is the same as real data. Although accuracy is a great evaluation method that intuitively represents the performance of a model, evaluating the performance using only the accuracy value is limited because the performance of the model can be distorted depending on the data composition. The formula for the accuracy is Equation (1).

(1) $$Accuracy=\frac{TP+TN}{TP+TN+FP+FN}$$

TP denotes a true positive, FP denotes a false positive, FN denotes a false negative, and TN denotes a true negative. Precision is the ratio of true data among the data predicted to be true by the learning model. The formula for the precision is Equation (2).

(2) $$Precision=\frac{TP}{TP+FP}$$

The recall is the ratio of the data predicted to be true by the learning model to the true data. The formula for the recall is Equation (3).

(3) $$Recall=\frac{TP}{TP+FN}$$

The F1-score is the harmonic mean of precision and recall and is used when data imbalance is significant; the higher the score, the better the model. The formula for the F1-score is Equation (4).

(4) $$F1-score=2\times \frac{Precision\times Recall}{Precision+Recall}$$

The dataset exhibited a severe class imbalance, particularly with the dominance of Level 0 and Level 1 samples. To address this, we evaluated both macro-averaged and weighted-averaged metrics. While weighted metrics reflect overall reliability by accounting for class frequencies, macro-averaged metrics treat all classes equally to highlight performance on minority classes.

(5) $$\mathrm{Macro}-F1=\frac{1}{K}\sum_{k=1}^{K}F1_{k}$$

(6) $$\mathrm{Weighted}-F1=\sum_{k=1}^{K}\frac{n_{k}}{N}F1_{k}$$

Furthermore, we introduced the Geometric Mean (G-mean), defined as the geometric mean of recall values across all classes. This metric was specifically selected for its high sensitivity to the misclassification of any single minority class.

(7) $$G-mean=\sqrt[{K}]{\prod_{k=1}^{K}Recall_{k}}$$

The performance of each model was evaluated by comprehensively considering the above evaluation indicators.

### 3.7. Computational Settings

All numerical experiments were conducted on an Apple M1 MacBook equipped with 16GB of unified memory. Deep learning models were implemented using PyTorch V2.6.0, utilizing the Metal Performance Shaders (MPS) backend for hardware accelerated training on macOS. In addition, traditional machine learning algorithms were executed via scikit-learn in a Python 3.9 environment.

### 3.8. Interpretability Analysis

To enhance the clinical interpretability of the top-performing model, this study employed SHAP (Shapley Additive exPlanations) analysis. We selected XGBoost for its optimal balance between classification performance and computational efficiency. To interpret the model, a Tree Explainer was applied to quantify the contribution of each of the 15 independent variables to the final activity level classification. This approach allows for a transparent understanding of how physiological, demographic, and temporal features influence the model’s decision-making process, moving beyond simple accuracy metrics.

### 3.9. Ablation Study Design

To clarify the practical contribution of clinical features, we compared two experimental configurations. Model A included 10 features (physiological, demographic, and temporal variables), while Model B integrated all 15 features, adding clinical phenotypes such as drinking, smoking, HTN, DM, and DL.

This comparison was performed using the top-performing models—XGBoost, Hard Voting, RNN, and GRU—to evaluate whether clinical data yield statistically significant improvements in accuracy and F1-score. Ultimately, this systematic analysis demonstrates that clinical context is a prerequisite for transforming raw physiological signals into actionable medical insights.

## 4. Results

This study suggested machine learning algorithms for classifying human activity levels by applying Python 3.8 and the Scikit-learn library. Section 4.1 summarizes the performance evaluation results and optimal parameters for sixteen algorithms. These models were designed to classify the physical activity status based on patients’ step counts and heart rate data over time. From this comprehensive evaluation, the optimal algorithm was identified and selected for further analysis. Section 4.2 presents the prediction performance evaluation when new patient data are input into the final machine learning models.

### 4.1. Performance of Models

The lifelogs used for learning were collected from 182 patients over roughly two months, with heart rate, step count, and calorie consumption measured every 15 min. In this study, sixteen algorithms were evaluated, and the performance evaluation results and optimal parameter values for each model are summarized in Table 3. Accuracy, recall, precision, and F1-score were used as evaluation metrics.

Statistical validation of these models demonstrated exceptional stability; for instance, the top-performing XGBoost model achieved a mean cross-validation (CV) score of 0.9449 with a remarkably low standard deviation of 0.0008. This statistical consistency confirms that the model’s high performance is not an artifact of specific sample selection but a robust characteristic across the entire dataset. While standard accuracy metrics present an optimistic view exceeding 94.4% for top-performing models, the introduction of macro-averaged metrics and G-mean revealed significant performance gaps between traditional machine learning and deep learning approaches, as summarized in Table 4. Notably, the GRU model failed to capture the extreme minority class (Level 4), resulting in a G-mean of 0.000 (Table 3). In contrast, while the RNN model achieved a macro F1-score of 0.8480 and a G-mean of 0.8016, its sensitivity to high-intensity activities remained slightly lower than that of the XGBoost model, which maintained the highest G-mean of 0.8052. Conversely, XGBoost and Hard Voting maintained a G-mean above 0.8010 and a macro F1-scores near 0.852, demonstrating superior capability in detecting minority classes despite the severe imbalance.

Since the differences in overall accuracy among the top-performance models were minimal (less than 0.1%), the final selection of XGBoost was based on a comprehensive evaluation of clinical utility and minority class detection. Among the top performers, XGBoost was identified as the most suitable model for clinical deployment as it provided an optimal balance of high classification performance—specifically for high-intensity activity levels—computational efficiency, and clinical robustness.

### 4.2. Prediction for Actual Patient Status

**Figure 6:**
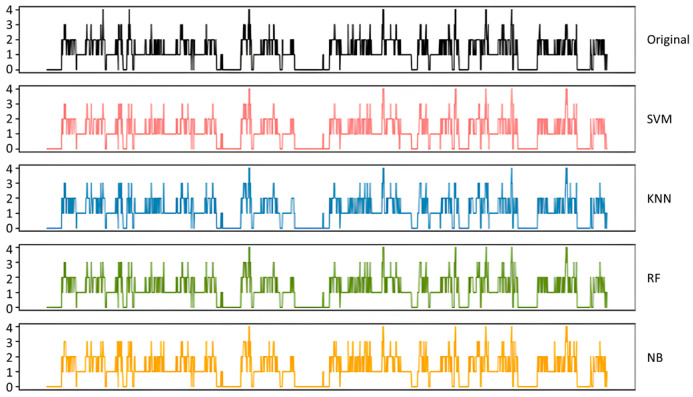
Test results of the final models.

XGBoost, Hard Voting, RNN, and the GRU were identified as the top-performing models for classifying human activity levels in real clinical scenarios. The parameters summarized in Table 3 were applied to each model. These machine learning models were applied to the new patient lifelog, including the heart rate and step count, and the results are shown in Figure 6. The results showed that the final machine learning models could classify the human activity level of a patient with an accuracy of over 93%.

Table 5 provides a comprehensive breakdown of classification performance across all five levels for the RNN model. While Class 0 achieved a perfect F1-score of 1.00 due to its deterministic definition, the model also maintained high performance for intermediate levels (Classes 1 and 2). The more modest scores for high-intensity activities—specifically Class 3 (Recall = 0.59) and Class 4 (Recall = 0.64)—reflect the inherent physiological noise in patient data. This further proves that the overall high accuracy is not artificially inflated but represents a realistic clinical evaluation of patient activity patterns.

### 4.3. Interpretability Analysis with SHAP

**Figure 7:**
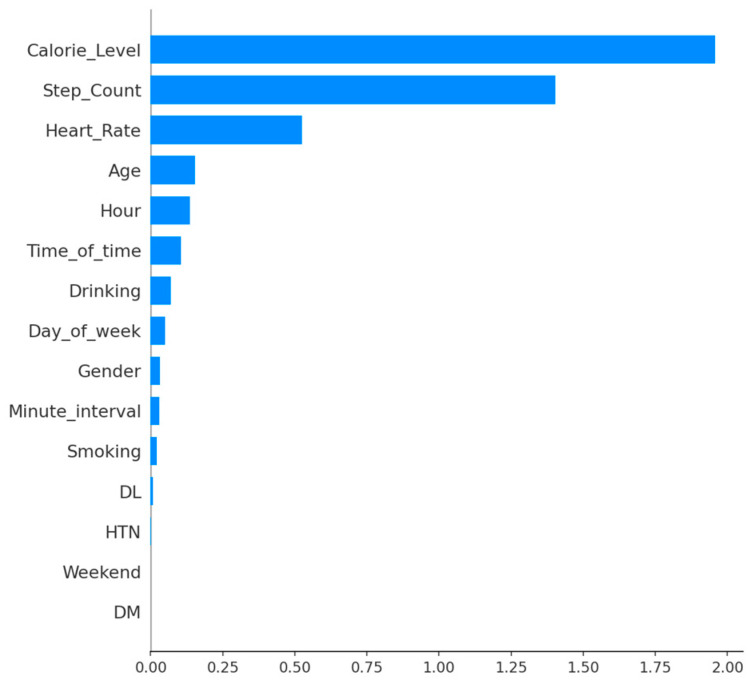
SHAP feature importance results of XGBoost.

The results of the SHAP analysis on the XGBoost model showed that lifelog variables were the most significant predictors of human activity levels. As indicated by the feature importance ranking, calorie level (1.957), step count (1.403), and heart rate (0.525) had the highest mean absolute SHAP values, representing most of the model’s predictive ability, see Figure 7.

While demographic factors like age (0.153) and temporal variables such as hour (0.136) contributed modestly, clinical parameters—including DM and weekend status—had minimal effect on the model’s predictive output. The results of this study suggest that high-frequency physiological lifelogs have a more immediate impact on activity intensity classification than static clinical phenotypes. However, including these clinical metrics remains essential for patient-specific frameworks to account for individual baseline physiological differences. By prioritizing this interpretable architecture, the proposed framework allows medical practitioners to move beyond ‘black-box’ predictions and make informed clinical decisions based on identifiable physiological contributors.

### 4.4. Ablation Study Results: Contribution of Clinical Features

The ablation study results on the test set demonstrated that the integration of clinical features (Model B) led to improved performance in ensemble-based machine learning models. XGBoost showed an increase in accuracy from 0.9440 to 0.9456 and F1-score from 0.9429 to 0.9447. Hard Voting similarly improved its accuracy from 0.9438 to 0.9452. In contrast, the impact on deep learning models was inconsistent; while RNN’s performance slightly declined from 0.9466 to 0.9447, the GRU showed a minor improvement from 0.9433 to 0.9440. These results indicate that while clinical data enhance the predictive power of ensemble-based ML models, its impact on sequential deep learning architectures varies depending on the specific model structure. Furthermore, although the SHAP analysis in Section 4.3 suggested that clinical variables had minimal individual impact on predictive magnitude, these ablation results confirm that integrating such phenotypes is a prerequisite for contextualizing raw physiological signals. This suggests that clinical feature provide a critical baseline context that, while subtle in feature importance rankings, helps stabilize and refine the classification of human activity levels in ensemble methodologies, see Table 6.

## 5. Discussion

The research proposes a machine learning-based framework for classifying physical activity into five different intensity levels, using wearable-derived lifelog data. Combining heart rate and step count metrics led to the creation of several advanced classifiers, including XGBoost and Hard-Voting ensembles, as well as deep learning models like the CNN and LSTM. These classifiers consistently achieved over 93% accuracy, demonstrating their reliability. Notably, while the premier models surpassed 94% in overall accuracy, our extended metric analysis highlighted that the deep learning architectures (RNN, GRU) were overly biased toward majority classes (Levels 0 and 1), resulting in a G-mean of 0 due to their failure in detecting high-intensity activities (Level 3). In contrast, well-tuned ensemble machine learning models, particularly XGBoost, proved highly robust to this inherent imbalance, reliably capturing minority class features and achieving a G-mean of 0.805. This superior performance of ensemble models is further supported by the ablation study, which demonstrates that integrating clinical features (Model B) consistently enhances the predictive power of XGBoost and Hard Voting compared to physiological-only models (Model A). This suggests that while physiological signals capture immediate intensity, clinical phenotypes provide the necessary baseline context for personalized classification. The handling of Level 0 prevented false sedentary assignment, and the ablation and stratified results support robustness across various patient conditions. The robustness of our findings is further reinforced by the statistical equivalence between the included and excluded patient groups. The lack of significant differences in demographic and clinical variables mitigates concerns regarding selection bias, confirming that the data refinement process focused solely on signal quality without compromising the clinical representativeness of the cohort.

The key contributions of this study are as follows. First, unlike earlier studies that mainly focused on basic activity metrics (such as daily step count or calorie consumption), this study shows that a more advanced classification of physical activity levels can be achieved by combining heart rate and step count data. Second, we introduce a dedicated ‘not-worn’ (Level 0) category to address the ‘zero-signal ambiguity’—a critical yet often overlooked challenge in human activity recognition (HAR). By clearly distinguishing device non-use from physiological inactivity, this approach effectively reduces ‘compliance bias’ and prevents the ‘phantom sedentary’ problem, where non-wear periods are incorrectly labeled as sedentary behavior. This improvement greatly boosts the reliability of patient adherence metrics, offering a stronger data foundation for clinical decision support systems (CDSS) in managing chronic conditions. Third, the comparative analysis of various machine learning techniques, including traditional ensemble and deep learning models, determines the optimal model for human activity classification.

Direct quantitative comparisons with state-of-the-art (SOTA) models trained on public benchmarks, such as UCI HAR or MHealth, were deliberately omitted in order to prioritize domain-specific optimization. Public datasets characteristically encompass laboratory-controlled activities undertaken by healthy cohorts, which are inadequate in capturing the clinical noise and physiological complexities intrinsic to comorbid patient populations. By prioritizing clinical utility over incremental gains in accuracy on standardized data, this study developed a robust classification framework integrating specific clinical phenotypes. This patient-centric architecture enhances interpretability for practitioners, fulfilling a critical requirement for effective medical decision-making.

Although the findings are encouraging, there are limitations. To ensure optimal data quality and reduce noise or variability caused by different sensor technologies and measurement protocols, this study intentionally used a single model: the Fitbit Inspire 2. Keeping a consistent data collection platform allowed the focus to remain on integrating clinical features and optimizing classification algorithms. However, we acknowledge that this approach limits the generalizability of the results across various wearable platforms.

Future studies should replicate the findings with various brands and models of wearable devices to ensure generalizability. The duration of the study was limited (approximately two months), which made it impossible to assess changes in activity patterns over the long term. Specifically, this relatively short timeframe may not fully capture seasonal variations or the long-term stability of physical activity patterns, which are critical for understanding behavioral shifts over extended periods. Therefore, future research with extended data collection periods may observe changing activity patterns over a longer time. Furthermore, while this study analyzed detailed characteristics of 182 participants with clinical backgrounds such as hypertension and diabetes, the cohort primarily consisted of relatively healthy adults without acute complications. Consequently, further research is needed to determine the specific impact of these comorbidities in more advanced disease stage. This skewed distribution may have limited the model’s ability to fully capture clinical variations in the dataset. However, from a clinical methodology perspective, integrating these variables is a prerequisite for contextualizing raw physiological signals into actionable medical insights. Therefore, incorporating such clinical data is crucial for improving the clinical relevance of HAR models and supporting the development of robust decision support systems tailored to individual patient health profiles. Future studies should investigate whether these models would also hold in the case of specific disease groups, such as patients with cardiovascular diseases or diabetes. In the future, the classification models presented in this work can be extended to real-time health monitoring and early anomaly detection. The ensemble approaches using high-performance XGBoost and voting-based classifiers have the potential to be used in clinical decision support systems. Adding extra body signals like blood pressure, body temperature, and sleep signals, etc. in the prediction can further enhance the predictive performance for better health assessment.

Results suggest that an advanced feedback technique based on machine learning should be developed for alerts when abnormal activities are detected. In the future, we should look into using these models in clinical applications and digital health platforms. This could lead to better health prediction accuracy.

## 6. Conclusions

This study proposes machine learning algorithms to classify patient physical status into five activity levels using lifelog data, including heart rate and step count, integrated with patient demographics and clinical information. Using comprehensive data from 182 patients comprising 15.72 million data points, both traditional machine learning (12 models) and deep learning (4 models) approaches were systematically evaluated, achieving over 94% classification accuracy across top-performing models.

The data used in the machine learning and deep learning models were collected using a Fitbit, a representative wearable device. Lifelogs were recorded over two months for a total of 182 participants who participated in the study and did not have specific medical conditions. The dataset included 15 engineered features, covering basic physiological signals, temporal patterns, aggregated time-series characteristics, and comprehensive patient demographics, thereby addressing the challenge of integrating multimodal healthcare data. The Fitbit device recorded heart rate data at a rate of once per minute and measured steps every 15 min. Calorie consumption was estimated at 15-min intervals, primarily based on assessments of the user’s physical attributes. The physical status of the subjects was categorized into four levels, based on their calorie consumption. Consequently, the data were pre-processed into an integrated 15-min format to improve the quality of the learning process. A new level (Level 0 [not worn]) was introduced, considering situations where the device was unworn or had battery issues. The input dataset included heart rate and step count data at 15-min intervals, while the output dataset classified human activity levels into five categories at the same intervals. Of the total data, 80% was used for training (12.58 million samples) and 20% for testing (3.14 million samples), with participant-wise splitting to prevent data leakage. The pre-processed data were applied to various machine learning models, including support vector machines (SVMs), k-nearest neighbors (KNNs), random forest (RF), naïve Bayes (NB), Hard Voting, Soft Voting, gradient boosting machines (GBMs), and XGBoost, as well as decision trees, logistic regression, extra trees, and AdaBoost, along with deep learning models. The best models were identified through 5-fold cross-validation and hyperparameter tuning. The system’s performance was assessed using several metrics, including accuracy, recall, precision, and the F1 score. The evaluation identified a high-performance group of models, with the top four reaching accuracy scores between 94.40% and 94.56%. Among these, XGBoost, Hard Voting, RNN, and the GRU were recognized as the top-performing models.

The classification models proposed in this study can be used in the healthcare field to define the human activity status using only the heart rate and step count of patients. Moreover, it can be used to analyze and predict the impact of lifelogs on the human body.

## Figures and Tables

**Table 1:** Independent variables.

Category	Feature Name	Value/Statistics
Demographic	Age	Mean 35 (20–63)
	Gender	F: 123/M: 59
Lifelog	Heart Rate (HR)	Continuous
	Step Count	Count
	Calorie Level	Continuous
Clinical	Drinking	Yes: 115/No: 67
	Smoking	Yes: 10/No: 172
	Hypertension Status	Yes: 12/No: 170
	Diabetes Mellitus Status	Yes: 14/No: 168
	Dyslipidemia Status	Yes: 20/No: 162
Temporal	Hour	Integer
	Minute	Categorical (15-min interval)
	Time of day	Morning (25.6%), Afternoon (24.0%), Evening (24.0%), Night (26.4%)
	Day of Week	Mon, Tue, Wed, Thu, Fri
	Weekend	Binary

**Table 2:** Baseline characteristics: included vs. excluded training candidates.

Variable	Included	Excluded	Test	*p*-Value	Effect Size
Age (years)	34.1 ± 7.4	28.3 ± 5.0	Mann– Whitney U	0.064	Cohen’s d = 0.798
Sex (Male, %)	37.5%	50.0%	Fisher’s exact	0.666	Cramér’s V = 0.020
Heart rate (bpm)	62.1 ± 9.6	22.8 ± 16.1	Mann– Whitney U	<0.001	Cohen’s d = 3.743
Step count	102.0 ± 31.4	46.6 ± 33.5	Mann– Whitney U	<0.001	Cohen’s d = 1.754
Calorie level (kcal)	4.4 ± 1.3	2.0 ± 1.4	Mann– Whitney U	<0.001	Cohen’s d = 1.859

**Table 3:** Machine learning performance evaluation and optimal parameters in each model.

Model	Parameters	Accuracy	Precision	Recall	F1-Score
XGBoost	n_estimators: 200, learning_rate: 0.1, max_depth: 5, subsample: 0.9	0.9456	0.9444	0.9456	0.9447
Hard Voting	-	0.9452	0.9439	0.9452	0.9440
RNN	Hidden: 128-64-35, MLP: 5 layers, Optimizer: AdamW, Batch Size: 256	0.9447	0.9443	0.9447	0.9435
GRU	Hidden: 128-64-32, MLP: 5 layers Optimizer: AdamW, Batch Size: 256	0.9440	0.9425	0.9440	0.9428
CNN	Filters: 64-128-256, Kernel: 3, MLP: 5 layers, Optimizer: AdmaW, Batch Size: 256	0.9439	0.9422	0.9439	0.9425
LSTM	Hidden: 128-64-32, MLP: 5 layers, Optimizer: AdamW, Batch Size: 256	0.9438	0.9423	0.9438	0.9427
Soft Voting	-	0.9444	0.9431	0.9444	0.9432
Gradient Boosting	n_estimators: 200, learning_rate: 0.1, max_depth: 5, subsample: 0.9	0.9442	0.9428	0.9442	0.9432
Decision Tree	max_depth: 20, min_samples_split: 5, min_samples_leaf: 2	0.9401	0.9384	0.9401	0.9386
Random Forest	n_estimators: 200, max_depth: 20, min_samples_split: 5, min_samples_leaf: 2	0.9386	0.9369	0.9386	0.9359
SVM	C: 10, gamma: 0.01, kernel: ‘rbf’	0.9375	0.9351	0.9375	0.9351
Logistic Regression	C: 1.0, penalty: ‘l2’, max_iter: 1000, random_state: 42	0.9352	0.9324	0.9352	0.9327
Extra Trees	n_estimators: 100, max_depth: 30, min_samples_leaf: 4, random_state: 42	0.9312	0.9293	0.9312	0.9277
Naïve Bayes	var_smoothing: 1 × 10^−9^	0.9109	0.9140	0.9109	0.9106
KNN	n_neighbors: 5, weights: ‘distance’, metric: ‘Euclidean’	0.8871	0.8826	0.8871	0.8813
AdaBoost	n_estimators: 100, learning_rate: 0.1, random_state: 42	0.7194	0.6529	0.7194	0.6442

**Table 4:** Model performance comparison via advanced metrics.

Model	Accuracy		F1-Score		G-Mean
	Macro	Weighted	Macro	Weighted	
XGBoost	0.8241	0.9456	0.8521	0.9447	0.8052
Hard Voting	0.8208	0.9452	0.8497	0.9440	0.8010
RNN	0.8220	0.9447	0.8480	0.9435	0.8016
GRU	0.7347	0.9440	0.7104	0.9428	-

**Table 5:** Quantitative performance comparison across all activity classes (RNN model).

Class	Activity Level	Precision	Recall	F1-Score
#0	Not worn	1.00	1.00	1.00
#1	Sedentary	0.96	0.97	0.96
#2	Lightly active	0.89	0.91	0.90
#3	Fairly active	0.75	0.59	0.66
#4	Very active	0.84	0.64	0.72

**Table 6:** Comparison of model performance in ablation study.

Model	Accuracy		F1-Score	
	Model A	Model B	Model A	Model B
XGBoost	0.9440	0.9456	0.9429	0.9447
Hard Voting	0.9438	0.9452	0.9425	0.9440
RNN	0.9466	0.9447	0.9461	0.9435
GRU	0.9433	0.9440	0.9402	0.9428

## Data Availability

For privacy reasons, the raw lifelog data that has PII cannot be shared. Researchers interested in accessing the anonymized dataset for research purposes should contact the corresponding author (parkkc07@tukorea.ac.kr) and complete a data use agreement.
